# Tincture of Thapsus

**Published:** 1879-01

**Authors:** James I. Tucker


### ARTICLE VIII.

*Tincture of Thapsus.*

As an efficient remedy for functional derangement of the uropoëtic system, a condition whose organic cause eludes analysis, I have for some time been in the habit of prescribing the tincture of thapsus bursa pastoris, or shepherd's purse, a genus of cruciferous plants. I treat with it not only hæmaturia, for which, on account of its astringent properties, it was formerly used, but cases of renal and cystic derangement very various in their nomenclature; but always this side of any considerable destructive lesion. I desire to draw to it the attention of the profession, because I believe it is a remedy not generally known. It has long been a favorite remedy with the so-called "eclectics," whose leading pharmacist first called my attention to it.

DR. JAMES I. TUCKER.

---

TREATMENT OF WHOOPING COUGH BY TINCTURE OF MYRRH. By Campardon.)

Whooping cough yields easily and rapidly to the alcoho c tincture (15 drops to the tablespoonful) in quinine wine or Vichy water. A tablespoonful every hour or two. This treatment in no way interferes with the appropriate remedies for the tracheobronchitis or the pulmonary congestion.—*Bull. Thér.*

